# Characterization of the acetabular labrum articular surface and its translation into biomimetic graft design

**DOI:** 10.3389/fbioe.2026.1749908

**Published:** 2026-04-07

**Authors:** Matthias X. T. Santschi, Stephanie Huber, Lukas Bienz, Simon Künzli, Casimir Schwank, Jess G. Snedeker, Michael Leunig, Stephen J. Ferguson

**Affiliations:** 1 ETH Zurich, Institute for Biomechanics, Zurich, Switzerland; 2 University Hospital Balgrist, University of Zurich, Zurich, Switzerland; 3 Department of Hip and Knee Surgery, Schulthess Clinic, Zurich, Switzerland

**Keywords:** electrospinning, labrum, melt electrowriting (MEW), tissue engineering, tribology

## Abstract

**Background:**

The acetabular labrum contributes to hip joint stability and lubrication, yet its articular surface properties remain poorly characterized. Understanding and replicating these surface features is critical for developing functional labral grafts.

**Methods:**

Native bovine labra were analyzed to assess surface microstructure, lubricin distribution, local stiffness, and coefficient of friction (COF). Scanning electron microscopy, immunohistochemistry, micromechanical indentation, and pin-on-disc testing were employed, with cartilage and meniscus samples as controls. In parallel, hybrid scaffolds combining melt electrowriting (MEW) microgrids with electrospun (SES) nanofibre caps were fabricated. These constructs were structurally and mechanically evaluated by SEM, T-peel adhesion tests, tensile testing, and pin-on-disc friction measurements for comparison.

**Results:**

The bovine labrum exhibited a dense nanofibrillar surface (∼70 nm fibres), abundant lubricin, and a low COF (0.15 ± 0.03), significantly lower than cartilage or meniscus. Trypsin digestion depleted lubricin, increasing COF and reducing local stiffness. Biomimetic MEW-SES constructs demonstrated successful nanofibre capping (∼0.83 µm fibres) over MEW grids (∼16.5 µm fibres). Adhesion between layers was moderate (3–4 mN/mm) and independent of MEW spacing. Nanofibre capping enhanced tensile modulus in dense MEW grids from 2.292 to 3.261 MPa and significantly reduced COF from 0.203 to 0.117, values within the physiological range of native tissue.

**Conclusion:**

The acetabular labrum possesses unique tribological properties that can serve as a blueprint for graft design. Hybrid MEW-SES constructs replicate key structural and functional features, providing a promising approach toward engineered labral grafts. Future work should integrate biological evaluation and long-term tribological testing under physiologic conditions.

## Introduction

Robust tribological properties of articulating surfaces are essential for preserving joint function. In the hip joint, the femoral head articulates with a complex surface comprising the acetabular cartilage and the inner surface of the acetabular labrum. While articular cartilage has been studied extensively with respect to its microstructure, lubrication, and mechanical behavior (Athanasiou et al., 1991; Bhosale and Richardson, 2008; Buckwalter, 1998), the surface properties of the acetabular labrum remain largely underexplored (Petersen et al., 2003; Basso et al., 2020).

Articular surfaces typically feature a fine fibrillar mesh oriented parallel to the joint surface, combined with the presence of lubricin and other boundary lubricants (Zhang et al., 2012; Chan et al., 2010). These surface factors are key to providing low-friction articulation, together with mechanisms such as interstitial fluid pressurization and fluid film lubrication (Krishnan et al., 2004; Wu and Ferguson, 2017). While the acetabular labrum is recognized for its sealing effect on the hip joint capsule, contributing to synovial fluid pressurization and joint stability (Ferguson et al., 2000; Philippon et al., 2014; Nepple et al., 2014), its intrinsic articular surface features may themselves contribute to joint lubrication.

Previous work has demonstrated that the bovine acetabular labrum possesses a nanofibrous surface mesh, abundant lubricin deposition, low permeability, and a coefficient of friction even lower than that of cartilage or meniscus (Zhang et al., 2012; Ferguson et al., 2001). These observations suggest that the labral surface has a unique role in maintaining lubrication and load distribution in the hip joint, beyond simply acting as a seal.

For regenerative strategies, recreating not only the bulk mechanics but also the surface features of labral tissue is essential (Grad et al., 2012; Gleghorn et al., 2007). Advances in tissue engineering have introduced melt electrowriting (MEW) and solution electrospinning (SES) as complementary scaffold fabrication technologies. Electrospinning can produce nanofibrous mats with extracellular matrix (ECM)-like features (Teo and Ramakrishna, 2006; Sell et al., 2010), while MEW enables precise deposition of microfibres with tunable porosity and defined architecture (Brown et al., 2011; Hrynevich et al., 2018). Combining these approaches can yield hybrid structures that mimic hierarchical tissues, including articular surfaces (Jungst et al., 2019; Dalton et al., 2020).

Thus, the characterization of the native acetabular labrum surface can serve as a blueprint for the engineering of biomimetic grafts. The present work integrates two approaches (Athanasiou et al., 1991): structural, mechanical, and tribological characterization of the bovine acetabular labrum surface; and (Bhosale and Richardson, 2008) development of nanofibre-capped MEW constructs to mimic these features, assessing their adhesion strength, tensile properties, and coefficient of friction.

## Methods

### Characterization of native labral tissue

#### Tissue harvest

For all measurements, hip and knee joints from young calves (356–585 days old cows, Metzgerei Angst Zürich) were dissected 2 days after slaughter. All specimens were derived from the food industry.

#### Presence of lubricin

Longitudinal and cross tissue slices were harvested from the thickest region of the labrum (n = 6), immediately fixed in 4% phosphate-buffered formaldehyde for 48 h at 4 °C, dehydrated in an ascending series of ethanol from 80% to 100% and isopropanol, and paraffin infiltrated (Logos J, Milestone, Sorisole, Italy) and paraffin embedded (Paraffin Embedding Station TES, Medite AG, Dietikon, Schweiz). 5 μm thick tissue sections were created using a microtome (HM 355S, Thermo Scientific, Reinach, Switzerland), air-dried at 37 °C, de-paraffinized in xylene, and rehydrated in a descending series of ethanol concentrations from 100% to 80% and distilled water. Enzymatic antigen retrieval was performed in a Tris-buffered Proteinase K solution (Proteinase K recombinant PCR Grade, 03 115 828 001, Roche Diagnostics GmbH, Mannheim, Germany) at a concentration of 20 μg/mL for 10 min at 37 °C. Endogenous Peroxidase activity was blocked with a Peroxidase Block (NovoLink™ Polymer Detection Kit, RE7140-K, Leica Biosystems Ltd, United Kingdom) for 5 min, and non-specific binding of primary and polymer reduced by application of a Protein Block (NovoLink™ Polymer Detection Kit, RE7140-K, Leica Biosystems Ltd, United Kingdom) for 5 min. Samples were incubated overnight at 4 °C in a purified mouse monoclonal IgG primary antibody to lubricin (Anti-Lubricin/Proteoglycan 4 clone 5C11, MABT400, EMD Millipore Corporation, USA) diluted 1:1000 in phosphate-buffered saline (PBS), followed by application of an anti mouse IgG containing Post Primary for 30 min (NovoLink™ Polymer Detection Kit, RE7140-K, Leica Biosystems Ltd, United Kingdom) and subsequent application of Novolink Polymer (NovoLink™ Polymer Detection Kit, RE7140-K, Leica Biosystems Ltd, United Kingdom) for another 30 min. The antibody complex was visualized by treatment with 3′3‐diaminobenzidine chromogen (NovoLink™ Polymer Detection Kit, RE7140-K, Leica Biosystems Ltd, United Kingdom) for 5min. Mounted sections (1900331, Shandon Mount Solution, Thermo Scientific, Reinach, Switzerland) were imaged using a high-resolution slide scanner (Slide Scanner Pannoramic 250, 3D Histech Ltd., Budapest, Hungary). Bovine cartilage tissue sections collected from the acetabulum were stained as a positive control, incubation with buffered saline alone and no primary antibody was used to ensure that staining is produced from detection of the antigen by the primary antibody.

#### Surface microstructure

In order to study the surface structure, thin labrum surface samples (n = 6) were fixed with a microwave-enhanced procedure involving a primary fixation in 2.5% glutaraldehyde (340855, Sigma-Aldrich, St. Louis MO, USA) and 4% paraformaldehyde (00380, Polysciences Inc., Warrington PA, USA) in 0.1M PIPES buffer (P1851, Sigma-Aldrich, St. Louis MO, USA) and a secondary fixation in 1% osmium tetroxide (0972A, Polysciences Inc., Warrington PA, USA) in double distilled water. The fixed tissue samples were dehydrated in an ascending series of ethanol from 50% to 100% and dried using a critical point dryer (CPD 931, Tousimis, Rockville MD, USA). The dried samples were coated with a 10 nm layer of Pt/Pd using a Metal Sputter Coater (CCU-10, Safematic, Rheintal SG, Switzerland) and imaged using a SU5000 SEM device (Hitachi, Tokio, Japan) at a non-disruptive acceleration voltage of 3 kV.

#### COF measurement

For the measurement of the coefficient of friction, cylindrical plugs with a diameter of 6 mm were harvested from at least two random locations of the articulating surface of the acetabular labrum and the meniscus (n = 5 each). Cartilage samples (n = 5) were removed from the opposite bovine femoral head with a scalpel and cut into discs with a diameter of 6 mm using a biopsy punch. The prepared tissue samples were then fixed in custom made sample holders, which allowed the tissue to be rehydrated in PBS overnight at 4 °C. The COF was measured on the following day.

**FIGURE 1:**
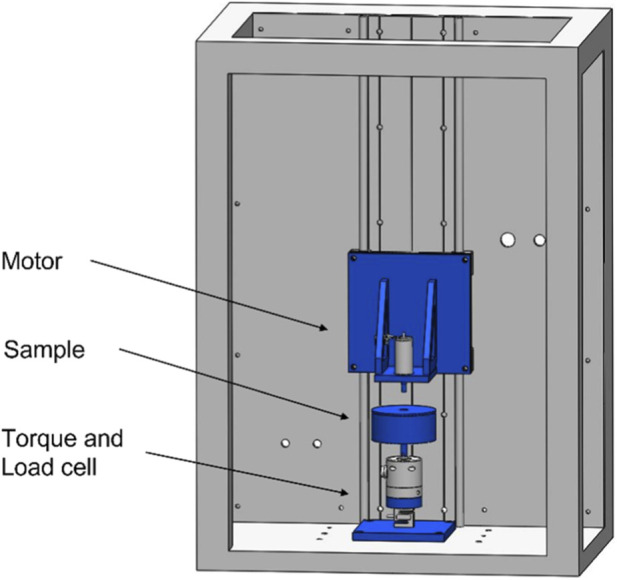
Pin-on-disc setup for the measurement of the coefficient of friction. The samples are submersed in a liquid environment within a sample holder. A motorized rotating flat-ended steel pin (r = 5.8 mm, Ra = 1.89 μm) acts as counter surface. Compressive load and torque are measured using sensors mounted below the sample holder.

Fully hydrated samples were placed in a liquid bath containing PBS to prevent dehydration. A flat ended stainless-steel pin with a measured surface roughness of Ra = 1.89 μm and a radius of r = 5.8 mm was rotated against the sample at 135 revolutions per minute (rpm). A defined contact pressure of 0.6 MPa was applied. The pressure was provided using a system of weights and counterweights on the pin holder, itself mounted to low-frition sliders, ensuring constant pressure throughout the entire experiment. The axis of rotation was chosen to be centered over the sample. Pressure and rotational speed at the outer edge of the pin were chosen to mimic contact pressure and the relative movement of the hip joint during a gait cycle. The liquid bath was mounted on a load cell (KD24S, 50 N, Transmetra, Switzerland) as well as a static torque sensor (DH15, ±0.005 Nm, Transmetra, Switzerland), to record the normal force $F_{N}$ and torque τ applied by the rotating stainless-steel pin. Figure 1 depicts a schematic representation of the testing setup.

The load and torque for comparative analysis were measured for 2 min and the coefficient of friction was calculated using Equation 1 below, which has been derived from the friction between two discs. In order to measure the development of the COF of acetabular labrum tissue over time, samples were measured at seven different time points, specifically 0, 1, 2, 5, 10, 20 and 30 min.

$$\mu =\frac{3\times \tau }{2\times F_{N}\times r}$$ (1)

#### Tissue digestion

Digestion with trypsin was used to mimic and study the impact of selective degradation of lubricin and structural damage of the articulating surface of the acetabular labrum (Ayala et al., 2025; Jay and Cha, 1999; Chan et al., 2010; Lawrence et al., 2015; Sun et al., 2008). Samples (n = 6) were harvested pairwise and prepared as mentioned before. After rehydration overnight, one of each pair was treated with Trypsin-EDTA derived from porcine pancreas (0.5%, Gibco, USA) for 30 min at room temperature, while the control was treated with PBS. After the treatment, all samples were thoroughly washed in PBS and the COF as well as the local stiffness were measured immediately. COF was determined using the previously described method. The COF was measured for 2 min and the value at 2 min was reported. The local stiffness of the articular surface was assessed using a micromechanical tester (FT-RS1002, Femtotools) equipped with a FT S1000 sensor covering a force range of ±1000 µN. A NiS sphere with a diameter of 232 µm was glued on the tip of the sensor and acted as a spherical indenter. Indentation was performed to a set threshold of 500 μN at an indentation speed of 10 μm/s. For each sample, 20 indentations at random positions were performed. The compressive modulus was calculated using the Hertz model with spherical indenter fitted to the recorded indentation force curves using custom MATLAB scripts.

#### Statistical analysis

For statistical evaluation of the COF of the different tissue types, an ordinary one-way ANOVA was performed followed by Tukey’s multiple comparisons test. To evaluate the difference between the native and degenerated state of the acetabular labrum, an unpaired t-test was performed. For the comparison of the local stiffness, a paired t-test has been performed. A significance level of p = 0.05 was defined.

### Fabrication and testing of biomimetic constructs

#### Melt electrowriting setup

All scaffolds were fabricated using a custom-built, in-house melt electrowriting device. The device consists of a vertically moving melt extrusion head positioned above a horizontally translating (X-Y) collector. The polymer in the melt head was kept in a 5 mL plastic syringe (Braun) connected to a 22 G blunt needle (Nordson). The polymer was kept molten in a heating jacket, which consists of an aluminum cylinder with two heating elements and a heat sensor (Mösch AG), connected to a proportional-integral-derivative (PID) feedback controller. Air pressure was applied to extrude the polymer melt through the needle. The collector was composed of a detachable, 1 mm thick, soda-lime glass plate on top of an aluminum plate. Attachment of the glass plate to the aluminum is facilitated by the application of negative pressure between the plates. Positive and negative high voltage were applied to the needle and the aluminum plate, respectively. The printing process was controlled through Mach4 (Newfangled Solutions, USA), a software commonly used for CNC machines. Furthermore, an enclosing climate control chamber (Parameter, USA) enables to control temperature and relative humidity. A custom-made Python tool was used to generate the G-codes for motion commands of the device enabling production of scaffolds with predefined architectures.

#### Melt electrowriting parameters

The PCL was kept at a melt head temperature of 80 °C and used for up to 7 days, before a new batch was loaded. An air pressure of 1 bar was applied to extrude the polymer melt. A positive high voltage of 3.8 kV and a negative high voltage of −1.5 kV was applied to the needle and the collector, respectively. The distance between the tip of the needle and the collector was set to 3 mm. High voltage and distance between the tip of the needle and the collector were increased by 10 V and 20 μm, respectively, for each layer, to compensate for the build height of the structure. The environment within the climate chamber was maintained at 21 °C and 41% relative humidity. For most fibres, the translating speed of the collector was chosen at 1.25 x CTS, where CTS is the critical translational speed allowing the fabrication of straight fibres.

#### Electrospinning

SES was performed on an electrospinning system manufactured by IME Technologies (Netherlands). The polymer solution used for SES was prepared as follows: PCL (Aldrich, USA) was dissolved in a 1:6 Methanol/NaCl:chloroform to a final concentration of 11% (w/v) PCL and 0.04% NaCl. The addition of NaCl has been shown to improve fibre diameter control, possibly through increased ionic polarisation of the solution, while leaving no residual NaCl in the final electrospun mat (Piyasin et al., 2019). The solution was left on a magnetic stirrer overnight and stored at room temperature prior to spinning. A blunt needle with an inner diameter of 0.6 mm was used as nozzle, which was placed 19 cm from a cylindrical collector with a diameter 90 mm. A low rotational velocity of 10 rpm was applied to the collector. A positive high voltage of 19 kV and a negative high voltage of −1 kV was applied to the nozzle and the collector, respectively. The polymer solution was extruded at a flow rate of 1.62 mL/h.

#### Nanofibre-capped MEW structures

A grid-like architecture for the MEW structures was selected, achieved by printing alternating layers of parallel lines, each arranged at a 90° angle to the previous layer. Scaffolds with 20 layers height and three different fibre spacings (1000 μm, 500 μm, 250 μm) were produced. The finished scaffolds were carefully removed from the collector glass plate and then cut into rectangular strips with dimensions of 40 mm length and 20 mm width and taped onto aluminium foil. For T-peel testing, one end of the specimens was covered with aluminium foil to create separated ends of both layers. Finally, the aluminium foil loaded with the samples was wrapped around the mandrel of the SES device and PCL was spun for 4 h onto the MEW scaffolds.

#### Structural characterization

Structural analysis of all sample types (n = 6 per type) was performed using a scanning electron microscope (SU5000, Hitachi, Japan) at the Scientific Center for Optical and Electron Microscopy (ScopeM), Zurich. Biopsy punches (Ø 6 mm) were used to punch out a part of the scaffold. These representative samples were subsequently mounted on SEM specimen stubs (Ø 10 mm) using a conductive carbon tape. Additionally, a Platinum/Palladium (PtPd) coating of 10 nm thickness was applied to ensure conductivity of the sample. Each structure was examined regarding fibre spacings and fibre thickness using ImageJ v1.53c. Fibre diameters and spacings were measured at the top two layers of the scaffold at 20 different spots per scaffold.

#### Sample preparation

The samples were cut out of the fibrous mat that had been spun onto the aluminium foil using a custom-made cutting tool composed of two parallel exchangeable microtome blades (S35, FEATHER, Japan). The aluminium foil was carefully removed before mechanical testing. All tests were performed on an Instron ElectroPulsTM E10000 dynamic testing machine with a 10 N load sensor (KD24s, Transmetra, Switzerland).

#### Adhesion tests

To measure the adhesion force, a T-peel test according to ASTM D1876 has been adapted to the given samples. For T-peel testing, the open ends from the samples described above were both clamped on a setup for tensile testing. Both ends were pulled apart at a peel rate of 0.5 mm/s. The end-position of the clamps was set to be 50 mm apart relative to the start point to ensure that the sample was peeled over almost its complete length. A total of 30 samples (N = 5 per scaffold type) were tested. Peel strength was expressed in terms of peel force divided by interface width. After completion of the test, all samples were imaged with a stereomicroscope (SZX 16, Olympus, Japan).

#### Mechanical tensile test

A uniaxial tensile test was performed under quasi-static conditions with a crosshead speed of 0.1 mm/s until a strain of 100% was reached. Five samples for each condition of the combined structures, five SES membranes which were cut from SES mats that were produced during fabrication of the combined scaffolds, and five uncapped MEW membranes per spacing were tested, yielding a total of 40 samples. All samples had the same size of 40 × 15 mm, guided by the ISO standard 527-3 for tensile testing of thin polymer films, which recommends an aspect ratio (L/W) ranging from 2 to 5. The thickness of the scaffold was measured with a Digital ABS Caliper 500-182–20 (Mitutoyo, Urdorf, Switzerland). The elongation of the specimen was measured using an optical extensometer (One-78 PT-200, Epsilon Technology Corp., USA). Force and displacement were converted to stress and strain through division by the cross-sectional area and clamp-to-clamp gauge length, respectively. The elastic modulus was determined by a linear fit of the stress-strain curve that followed the initial non-linear toe region.

#### Friction test

For the measurement of the coefficient of friction, cylindrical discs with a diameter of 6 mm were cut from MEW250 and MEWSES250 (n = 5 each). The samples were then fixed on custom made sample holders, and fully hydrated in PBS overnight at 4 °C. Fully hydrated samples were placed in a liquid bath containing PBS to prevent dehydration. To ensure comparability, the same setup used for the native labrum tissue was used here as well.

#### Statistical analysis

The obtained data was processed using a custom script in Jupyter notebooks (Python 3.8.8). All statistical analyses and graphs were produced with GraphPad Prism (v.3.2.0). For the statistical evaluation of the tensile modulus, an ordinary one-way ANOVA with multiple comparison has been perfomed. The obtained values for the COF of nanofibre capped vs. non-capped microfibre grid were compared using an unpaired T-Test. A significance level of p = 0.05 was defined.

## Results

### Native labral surface

**FIGURE 2:**
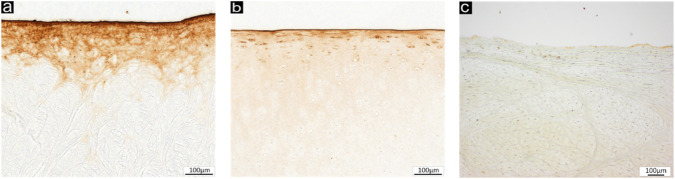
Representative immunhistochemically stained samples of **(a)** bovine cartilage derived from the acetabulum, **(b)** bovine acetabular labrum, both showing substantial lubricin at the articulating surface and **(c)** trypsin digested bovine acetabular labrum samples showing the removal of lubricin.

Immunohistochemical analysis demonstrated strong surface and subsurface expression of lubricin across all bovine labral samples, with staining extending up to 300 µm depth in some donors. In contrast, digested specimens showed a clear depletion of lubricin, confirming the effectiveness of the enzymatic treatment in removing boundary lubricants (Figure 2).

**FIGURE 3:**
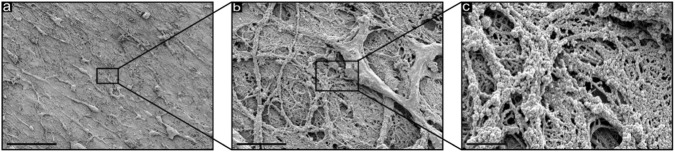
SEM images of the articular surface of the acetabular labrum. **(a)** Overview of the relatively smooth and flat surface, with some macroscopic structures visible. Scalebar = 50 μm. **(b)** Increased magnification revealing the surface consisting of nanofibers with some macroscopic structures. Scalebar = 5 μm. **(c)** High magnification revealing nanofibers in the range of 70 nm. Scalebar = 1 µm.

Examination of the surface microstructure by scanning electron microscopy (Figure 3) revealed a dense nanofibrillar mesh composed of fibres with a diameter of approximately 70 nm, occasionally forming thicker bundles protruding through the surface. No distinct preferential orientation of the fibres was observed.

**FIGURE 4:**
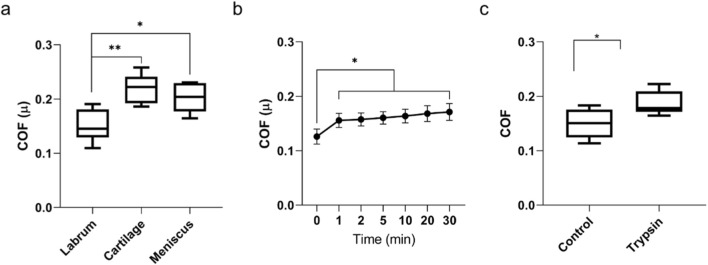
**(a)** Coefficient of friction of cylindrical samples derived from the bovine articular labrum, cartilage and meniscus (n = 5). **(b)** Temporal development of the coefficient of friction of labrum samples from 1 to 30 min (n = 5). **(c)** Comparison of the coefficient of friction between non-treated control samples and trypsin-treated samples (n = 5). Statistical significance is indicated as follows: p ≤ 0.05 as * and p ≤ 0.002 as **, respectively.

Tribological testing (Figure 4) showed that the coefficient of friction (COF) of the labrum surface was 0.15 ± 0.029, significantly lower than that measured for cartilage (0.22 ± 0.027) or meniscus (0.20 ± 0.026). A time-dependent increase in COF was observed, rising moderately from 0.13 at the onset of testing to 0.17 after 30 min. Trypsin digestion elevated the COF further to 0.19 ± 0.022. The mean local modulus of the native labral surface was found to be 115.7 ± 23.7 kPa. The modulus of the trypsin treated articular surface was significantly reduced (P < 0.0001) to 45.1 ± 32.3 kPa.

### Biomimetic constructs

**FIGURE 5:**
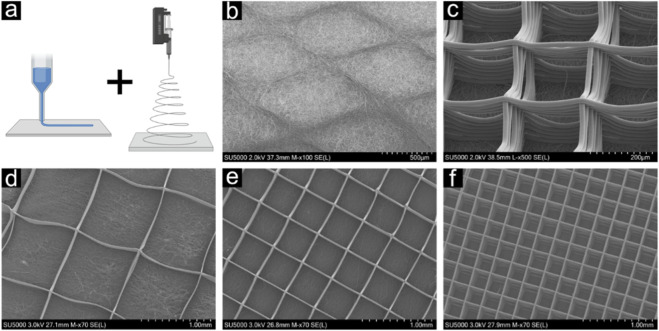
Structures obtained by **(a)** combining melt electrowriting (MEW) and electrospinning. **(b)** Articulating surface formed by electrospinning, spanning the underlying MEW layer. **(c)** Detail of the underlying MEW structure, viewed towards the nanofibre electrospun cap. Different MEW grid sizes were evaluated: **(d)** 250, **(e)** 500 and **(f)** 1000 µm.

Nanofibre capped microfibre grids were produced by performing SES on a previously fabricated MEW microfibre grid, which consisted of a total of 20 layers and fibre spacings of 250, 500 and 1000 µm. SEM analysis of the fibre morphology (Figure 5) revealed diameters of 0.828 ± 0.389 µm and 16.517 ± 1.421 µm for the electrospun nanofibre mesh and the melt electrowritten microfibre grid, respectively. While the main portion of the nanofibre grid was found to be spanning over the microfibre grid, only very few nanofibres penetrated into the spaces of the microfibre grid.

**FIGURE 6:**
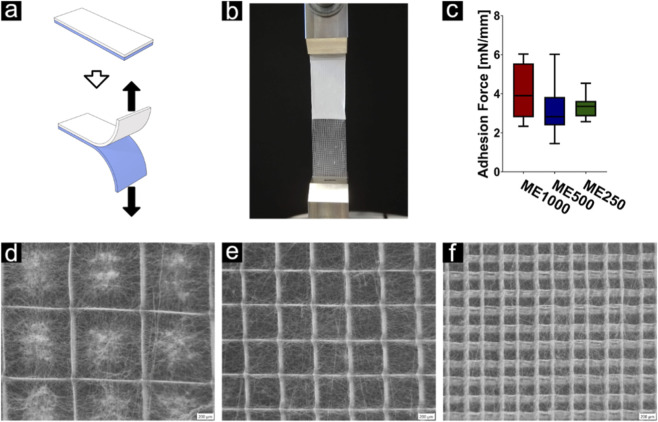
**(a,b)** T-Peel test performed to assess the adhesion strength between the (semi-translucent) microfibre grid and the (white) nanofibre cap. **(c)** The adhesion strength was found to be similar throughout all grid sizes: **(d)** 250, **(e)** 500 and **(f)** 1000 µm. Visual inspections of the MEW structures revealed nanofibre remnants, indicating adhesion between the two components.

A T-peel test was performed for each of the three constructs ME1000, ME500 and ME250 was performed, to compare the effect of fibre spacing of the underlying MEW structure on the adhesion force (Figure 6). The mean adhesion force for ME1000, ME500and ME250 were found to be 4.023 ± 1.377 mN/mm, 3.154 ± 1.264 mN/mm and 3.310 ± 0.567 mN/mm, respectively. No significant differences have been found between the three test groups. Visual inspections of the MEW structures revealed nanofibre remnants, as shown on the pictures taken with a stereomicroscope (Figures 6d–f).

**FIGURE 7:**
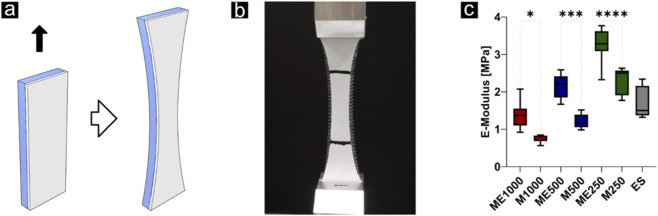
Quasistatic tensile tests **(a,b)** showed significantly increased E-Moduli when the microfibre MEW grid is capped with a nanofibre membrane (ME), compared to the non-capped MEW grid (M). **(c)** Only the capped 250 µm grid had a significantly higher E-Modulus, compared to the nanofibre membrane only. However, E-modulus values are normalised to specimen cross-sectional area. Statistical significance is indicated as follows: p ≤ 0.05 as *, p ≤ 0.001 as ***, and p ≤ 0.0001 as **** respectively.

Uniaxial quasistatic tensile testing was performed to assess the influence of the nanofibre cap on tensile properties (Figure 7). Tensile (Young’s) modulus has been analyzed. The tensile modulus for each group (n = 5) was found to be 1.378 ± 0.338 MPa (ME1000), 0.7684 ± 0.115 MPa (M1000), 2.16 ± 0.307 MPa (ME500), 1.212 ± 0.198 MPa (M500), 3.261 ± 0.417 MPa (ME250), 2.292 ± 0.367 MPa (M250) and 1.713 ± 0.432 MPa (ES). Significant differences between capped and their uncapped counterpart were found with p = 0.03, p = 0.0001 and p < 0.0001 for the MEW structures with fibre distances of 1000, 500 and 250 μm, respectively.

**FIGURE 8:**
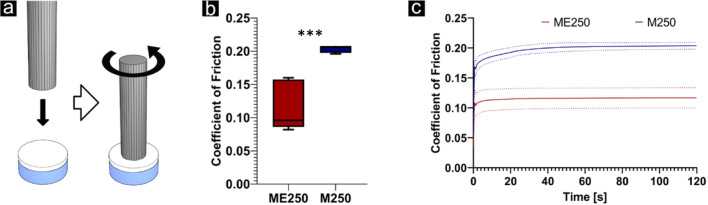
**(a)** A pin-on-disc setup was used to assess the coefficient of friction (COF) of nanofibre capped constructs and melt electrowritten microfibre grids. **(b)** The addition of the nanofibre cap (MEW250) resulted in a significantly reduced COF, compared to bare MEW scaffolds (M250), both with 250 µm grid spacing (p ≤ 0.001, shown as ***). **(c)** Both scaffold types exhibited a running-in behaviour similar to that of natural labrum tissue.

To measure the coefficient of friction on PCL structures against a rotating steel pin in PBS, and in addition to characterize the impact of a nanofibre mat on a microfibre grid with respect to the coefficient of friction, samples of ME250 and M250 were tested on a pin-on-disc setup for friction measurements (Figure 8). ME250 and M250 exhibited the most promising tensile properties. The COF for M250 and ME250 were found to be 0.2034 ± 0.006 and 0.1166 ± 0.037, respectively. A significant reduction of the COF was found in the group containing the nanofibre cap (p = 0.0009).

## Discussion

The acetabular labrum is increasingly recognized as more than a passive stabilizer of the hip joint; its surface structure and tribological function appear integral to maintaining joint lubrication and load distribution. In this study, we demonstrated that the bovine labrum surface is characterized by a dense nanofibrillar network with fibre diameters around 70 nm, abundant lubricin deposition extending several hundred micrometers into the tissue, similar to that observed in the meniscus (Peng et al., 2015), yet with exceptionally low frictional properties compared to cartilage and meniscus. These findings confirm earlier descriptions of the labrum as a highly specialized fibrocartilaginous tissue with unique surface properties (Petersen et al., 2003; Zhang et al., 2012) and extend them by providing quantitative tribological data. The observed coefficient of friction (0.15 ± 0.029) is consistent with reported values for articular cartilage (Athanasiou et al., 1991; Bhosale and Richardson, 2008), yet lower and more stable over time.

Trypsin digestion depleted lubricin and proteoglycans, resulting in increased friction and reduced local stiffness, in agreement with studies showing the central role of lubricin and boundary lubrication in articular surfaces (Chan et al., 2010; Krishnan et al., 2004). Trypsin has previously been used to selectively degrade lubricin in synovial fluid and at the surface of articulating tissues (Ayala et al., 2025; Jay and Cha, 1999; Chan et al., 2010; Lawrence et al., 2015), and this has lead to a reduction or elimination of boundary lubrication, increase of friction, and sub-surface damage to cartilage. Trypsin digestion has also been shown to dramatically increase the gliding resistance of tendons (Sun et al., 2008). While previous studies have demonstrated a beneficial role of mechanical stretching (Huber et al., 2022) and compression/shear (Huber et al., 2023) in stimulating the expression of PRG4 (lubricin) in labrum tissue, surgical samples from pathological joints exhibit lubricin depletion (Huzum et al., 2025).

The structural similarity of the labrum surface to other articular tissues such as cartilage and meniscus is notable. All share a superficial fibrillar layer of collagen oriented parallel to the articular surface (Basso et al., 2020; Gottardi et al., 2016), yet the labrum is distinguished by its low permeability and sealing effect on the joint space (Ferguson et al., 2000; Nepple et al., 2014). Together, these attributes suggest that the labrum contributes directly to lubrication via fluid pressurization and film retention (Wu and Ferguson, 2017), while its surface biochemistry enhances boundary lubrication. Thus, characterization of the labral surface offers valuable design cues for engineered grafts. Degenerated acetabular labrum samples exhibit pronounced ultrastructural disruption, including fibrillation, altered collagen organisation and surface discontinuities (Huzum et al., 2025), likely compromising labrum function.

To date, synthetic grafts for labrum repair have been mostly produced from foamed polyurethane (Tey-Pons et al., 2021). While these have been shown to improve contact pressure and restore labral sealing to some degree (Capurr et al., 2022), they do not reproduce the structure of the natural labrum or provide topological cues for cell growth. The present study explored the fabrication of biomimetic constructs by combining melt electrowriting (MEW) microfibre grids with solution electrospun (SES) nanofibre caps. The rationale for this hybrid approach derives from the complementary strengths of the two technologies: electrospinning produces nanofibres resembling the extracellular matrix but with limited porosity and poor cell infiltration (Teo and Ramakrishna, 2006; Sell et al., 2010), while MEW generates highly ordered microfibre scaffolds with defined pore architectures suitable for cell migration but lacking nanoscale surface features (Brown et al., 2011; Hrynevich et al., 2018). MEW scaffolds for meniscal repair have been proposed, however these exhibited tensile moduli in the range of only 100–500 kPa (Korpershoek et al., 2021). We have previously described multiscale MEW scaffolds with favourable mechanical properties, i.e., moduli over 10 MPa, and compatibility with primary labrum cells (Santschi et al., 2022), however these do not directly reproduce the unique structure of the articular surface. Hybrid fabrication has been proposed to address these limitations (Jungst et al., 2019; Dalton et al., 2020). Multi-scale, osteochondral scaffolds with depth-dependent zonal architecture utilizing SES and MEW layers achieved varying E-moduli, but from only 10–1000 kPa (Steele et al., 2022). In multi-scale bilayer MEW scaffolds, the addition of a thin surface tangential zone improved modulus values 2- to 3-fold, however only up to 500 kPa (Castilho et al., 2019). Gel infiltration has been suggested to improve the properties of MEW scaffolds for meniscus repair (Barceló et al., 2023a; Barceló et al., 2023b). Nevertheless, even after prolonged culture with cells, these also showed lower moduli values than those measured for the scaffolds in the present study. Relatively little is known about the tribological outcomes of combining MEW and SES. For the specific application, it was envisioned that a SES cap on the MEW grid could provide a biomimetic articular surface.

Our findings demonstrate that electrospun nanofibres (0.83 µm in diameter) consistently formed a capping layer over MEW fibres (16.5 µm in diameter) without significant penetration into the microgrid pores. This differs from approaches where MEW is deposited directly on electrospun mats (Jungst et al., 2019), and was thought to potentially produce more integrated fibre architectures. Adhesion between SES and MEW layers averaged 3–4 mN/mm and was independent of grid density, with delamination occurring within the SES layer. These values are comparable to previously reported adhesion strengths between electrospun mats (Bouchet et al., 2021; Ballarin et al., 2013), suggesting that SES–MEW interfaces can be mechanically stable but remain susceptible to internal failure of the nanofibre mat.

Tensile testing further revealed that the mechanical contribution of the nanofibre cap depended on MEW spacing. In sparse grids (1000 μm, 500 µm), the electrospun layer dominated, and the MEW contribution was minimal. By contrast, in dense grids (250 µm), the combined construct achieved significantly higher tensile modulus than SES alone. The values for SES-MEW approached those of, e.g., silk fibroin scaffolds previously described for meniscal repair (Warnecke et al., 2018), which demonstrated moduli slightly above 5 MPa, but without distinct surface properties. This suggests that MEW provides reinforcement only at a sufficient density, consistent with previous work highlighting the role of pore size and fibre spacing in determining scaffold mechanics (Hrynevich et al., 2018).

Most importantly, tribological testing showed that nanofibre capping reduced the COF of MEW constructs from 0.203 to 0.117, placing the values within the range of native labrum, cartilage and engineered articular tissue replacement materials (Li et al., 2010; Accardi et al., 2013). This reduction is consistent with prior findings that nanoscale surface topography improves synovial fluid retention and boundary lubrication (Wu and Ferguson, 2017). Interestingly, the COF remained stable over the test duration, in contrast to native cartilage, where values typically increase as fluid pressurization dissipates (Krishnan et al., 2004; Li et al., 2010). This suggests that the capped constructs may resist fluid loss and maintain lubrication, possibly due to enhanced micro-topography and surface hydrophilicity provided by the nanofibre layer. The values achieved in the present study compare favorably with those shown for the previously mentioned silk fibroin scaffolds, which approached but still exceeded the COF of natural meniscus by 3 – 4x, and also showed a pronounced increase of strain with prolonged loading, implying a loss of fluid pressurization (Warnecke et al., 2017). The frictional properties of the SES-MEW scaffold may be further enhanced by gel infiltration, where COF values as low as 0.04 have been demonstrated with electrospun membrane reinforced hydrogels (Chen et al., 2023).

Taken together, the results from native labrum characterization and biomimetic construct fabrication and characterization highlight both the opportunities and challenges of recreating articular surfaces. The labrum surface provides a blueprint of nanofibrillar architecture and lubricin-rich chemistry that underpins its low-friction function. Hybrid MEW–SES scaffolds represent a promising engineering strategy, capable of reducing friction to physiological levels and reinforcing tensile mechanics when appropriately designed. However, further optimization is needed to improve fibre integration, adhesion strength, and long-term stability under cyclic loading. Moreover, biological studies incorporating labral or chondrocyte populations will be required to assess ECM deposition, biocompatibility, and remodeling potential.

Future work should also address the translational gap between *in vitro* tribology and *in vivo* joint lubrication, which depends on the combined effects of synovial fluid constituents, dynamic loading, and biological adaptation. Nevertheless, the present findings provide a strong rationale for pursuing hybrid scaffold designs in labral graft engineering and establish the labrum surface as a valuable biological template for articular tissue replacement strategies.

This study has several limitations. The use of bovine calf labra may not fully represent adult human tissue, and friction testing against steel pins does not replicate native cartilage-on-labrum contact. Trypsin digestion, while effective in depleting lubricin, is non-specific and alters multiple extracellular matrix components. In the engineered constructs, electrospun fibres bridged rather than penetrated MEW pores, limiting integration, and adhesion failures occurred mainly within the electrospun layer, indicating a need for optimization. Tribological testing was restricted to short timeframes, and no biological studies were performed, leaving questions about cell interaction and ECM remodeling unresolved. Finally, *in vivo* lubrication depends on additional synovial and biological factors not captured in the present experimental setup.

## Conclusion

The bovine acetabular labrum exhibits a lubricin-rich nanofibrillar surface with low friction and unique tribological behavior. The coefficient of friction (COF) of the labrum surface was 0.15, significantly lower than that measured for cartilage or meniscus. Trypsin digestion increased COF. Hybrid MEW-SES scaffold constructs can replicate key aspects of natural labrum structure and function. MEW-SES constructs achieved physiological COF values of 0.12, and the addition of a SES mesh surface layer reinforced the tensile modulus significantly, compared to MEW alone. These findings provide a blueprint for developing functional labral grafts. Future work should include biological integration, long-term tribology under cyclic loading, testing against native cartilage and upscaling to native tissue geometry.

## Data Availability

The raw data supporting the conclusions of this article will be made available by the authors, without undue reservation.
